# Dr. Eugene S. Forman

**Published:** 1898-10

**Authors:** 


Dr. Eugene S. Forman, of Auburn, N. Y., U. of B. '70, died at his home, September 13, 1898, aged 51 years. His death was sudden, and it is announced was caused by apoplexy.

